# A Discussion Concerning the Inclusion of Variety Effect when Analysis of Variance is Used to Detect Differentially Expressed Genes

**Published:** 2007-06-15

**Authors:** Guri Feten, Are Halvor Aastveit, Lars Snipen, Trygve Almøy

**Affiliations:** Department of Chemistry, Biotechnology and Food Science, Norwegian University of Life Sciences, P.O.Box 5003, N-1432 Ås, Norway

**Keywords:** Hypothesis test, main and interaction effects, microarray experiment

## Abstract

In microarray studies several statistical methods have been proposed with the purpose of identifying differentially expressed genes in two varieties. A commonly used method is an analysis of variance model where *only* the effect of interaction between variety and gene is tested. In this paper we argue that in addition to the interaction effects, the main effect of variety should simultaneously also be taken into account when posting the hypothesis.

## 1 Introduction

The technology of DNA microarray gives us the opportunity to screen the expression levels for thousands of genes simultaneously. An introduction to this technology can be found in e.g. Nguyen et al. (2002). One of the important issues when measuring gene expression microarray is to search for genes which are Differentially Expressed (DE) in two different varieties. A gene is DE if the amount of mRNA is different in the two samples compared. The samples compared can be from different times of a biological process, different strains of a bacterium, or different conditions (e.g. treatment or disease).

Many statistical models and methods have been proposed in the search for DE genes. In this paper we will focus on the Analysis of Variance (ANOVA) model. This model for finding DE genes was introduced by Kerr et al.(2000, 2001, 2002), Wolfinger et al. (2001),and Kerr and Churchill (2001a and Kerr and Churchill (2001b). A commonly used hypothesis for identifying DE genes is to test merely the effect of interaction between variety and gene. We argue that in addition to the interaction effects the main effect of variety should be included simultaneously in the hypothesis. The latter hypothesis is briefly discussed by Black and Doerge (2002) and Craig, Black and Doerge (2003), but not as the main topic of their papers. The hypothesis introduced by Kerr et al. is widely spread, and is still popular, even after the introduction of the hypothesis with the main effect included. Due to this, we do a more thorough discussion on the two hypotheses and discuss the consequences of choosing one of them in preference to the other one.

The outline of the paper is as follows. In Section 2 we state the model and two different hypotheses for detecting DE genes, one based on the interaction effect and the other based on both the interaction effect and the main effect simultaneously. In addition, we discuss the differences, both with new arguments and with arguments taken from Black and Doerge (2002) and Craig et al. (2003). Both hypotheses are tested on an example in Section 3, and in Section 4 we summarize the results obtained.

## 2 The Model

In previously published papers concerning the problem of detecting DE genes, different authors have applied ANOVA-models (Kerr et al. 2000, 2001, 2002, Wolfinger et al. 2001, Kerr and Churchill, 2001a, 2001b, Craig et al. 2003). The model suggested in Kerr et al. (2000) (from now on denoted Kerr) is

(1) $$\begin{array}{l} y_{ijkg}=\mu +A_{i}+D_{j}+V_{k}+G_{g}\\ +{\left(AG\right)}_{ig}+{\left(VG\right)}_{kg}+{ɛ}_{ijkg}, \end{array}$$

where *y_ijkg_* is the expression level on the log scale for gene *g* of variety *k* measured on array *i* using dye *j*. Further, μ is the overall mean, *A_i_* is the effect of the *i*th array, *D_j_* is the effect of dye *j*, *V_k_* is the effect of the *k*th variety, *G_g_* is the effect of gene *g*, (*AG*)*_ig_* is the interaction effect between array *i* and gene *g*, (*VG*)*_kg_* is the interaction effect between variety *k* and gene *g*, and ε*_ijkg_* is an error term independently distributed as ε*_ijkg_* ~ *N*(0, σ^2^). In this model *D_j_*, *V_k_*, *G_g_*, and (*VG*)*_kg_* are assumed to be fixed, while *A_i_* and (*AG*)*_ig_* are commonly assumed to be random.

There are sixteen possible effects, included interactions of all orders. With a dye-swap experiment each of the sixteen effects is completely confounded with one other effect, hence one effect is only possible to estimate assuming the confounding effect is zero. A table showing the pairs of confounded effects in a dye-swap experiment can be found in Kerr.

Additional effects, such as the interaction between dye and gene, (*DG*)*_jg_*, could have been included in the model. However, these effects use degrees of freedom that should be used for estimating error. Omitting the effect of (*DG*)*_jg_* in the analysis of a dye-swap experiment will not alter the estimates of the other effects in the model, due to the orthogonality of the design.

The objective of the experiment is to identify genes that are differentially expressed in treatment and control samples. Previous papers state that genes with significant interaction between variety and gene are DE. Hence, the effect of interest in Model (1) is (*VG*)*_kg_* (Kerr et al. 2000, 2001, 2002, Wolfinger et al. 2001, Kerr and Churchill, 2001a, 2001b). According to these authors, a gene *g* is DE if and only if (*VG*)_1_*_g_* − (*VG*)_2_*_g_* is non-zero. For fixed effects the restriction ∑*_k_* _= 1_^2^(*VG*)*_kg_* = 0 is usually assumed, hence (*VG*)_1_*_g_* − (*VG*)_2_*_g_* ≠ 0 corresponds to (*VG*)*_kg_* ≠ 0. This gives the following statement:

### Statement 1

*A gene g is DE if and only if (VG)_kg_* ≠ 0.

The hypothesis of interest,

(2) $$H_{0g}:{\left(VG\right)}_{kg}=0,$$

is tested with a t-test for each gene separately.

In this paper we will show that the study of the difference (*VG*)_1_*_g_* − (*VG*)_2_*_g_* in some cases is not accurate enough, in fact it is not even correct, hence, Statement 1 is incorrect. If our interest is focused on whether a gene is DE in two varieties, we should rather examine the corresponding expected difference between the gene expressions in the two varieties. If we assume the restrictions ∑*_k_* _=1_^2^ *V_k_* = 0 and ∑*_k_* _=1_^2^(*VG*)*_kg_* = 0, this is given by

$$\begin{array}{l} E(\bar{y}.._{1g}-\bar{y}.._{2g})=(V_{1}-V_{2})+({\left(VG\right)}_{1g}-{\left(VG\right)}_{2g})\\ =2V_{1}+2{\left(VG\right)}_{1g}, \end{array}$$

where $\bar{y}.._{kg}=\frac{1}{2a}\Sigma_{i=1}^{a}\Sigma_{j=1}^{2}y_{ijkg}$ for *k* = 1, 2. A gene is DE if there is an effect of variety, either main effect, interaction effect, or both. This gives the following statement:

### Statement 2

*A gene g is DE if and only if (V_1_* *+ (VG)_1g_)* ≠ 0.

The hypothesis of interest,

(3) $$H_{0g}:V_{1}+{\left(VG\right)}_{1g}=0,$$

is carried out with a t-test for each gene separately. The two hypotheses are equal if and only if *V*_1_ = 0. The hypothesis in (3) is presented in Black and Doerge (2002) as an alternative hypothesis to the hypothesis in (2).

**Figure 1:**
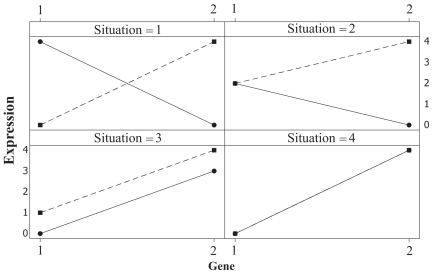
Different situations for main effect and interaction between variety and gene. Variety 1 (—–•—–) and Variety 2 (– –■– –).

The differences between Statement 1 and 2 are illustrated in Figure 1. According to both statements both genes in situation 1 are DE. In situation 2, Statement 1 claims both genes to be DE, while Statement 2 claims gene one to be Equally Expressed (EE) and gene two to be DE. In situation 3 both genes are EE according to Statement 1, while both genes are DE according to Statement 2. According to both statements both genes are EE in situation 4.

If only the interaction terms are used to test for DE genes, the conclusions are dependent on the choice of genes printed on the array. Adding a new gene to the array, or removing a gene from the array, may change the level of interaction, and hence the conclusions about which genes are DE.

A necessary assumption if Statement 1 should be preferred, is that the majority of the genes are equally expressed, and that differentially expressed genes, either up- or down-regulated, will balance each other out. Then there is no true effect of variety, and the corresponding term can be excluded from the hypotheses. However, differentially expressed genes occur more frequently than previously assumed (van de Peppel et al. 2003).

Both Black and Doerge (2002) and Craig et al. (2003) suggest that Statement 2 is to prefer when there is a true difference between the varieties averaged over all genes, while Statement 1 is to prefer when the variety effect is an artifact of the experimental process, however, this is usually difficult to decide. For dye-swap experiments the effect of variety and the interaction between array and dye are confounded, while for reference designs the effect of variety confounds with the effect of array, hence the effect of variety cannot be separated from experimental effects.

To get an idea about any possible effect of interaction between array and dye (or with reference designs, the effect of dye), a pilot study with self-self hybridization can be performed. Samples from one of the varieties are divided in two, and then labeled with Cy5 and Cy3, respectively. Hence there is no true variety effect, and any estimated effect is interaction of array and dye. If this interaction effect seems large, the hypothesis in (2) should be applied, while if the interaction effect seems small or ignorable, the hypothesis in (3) should be applied.

As also stated in Kerr (2003) a replacement of the term *V_k_* in Model (1) with the confounding interaction effect between array *i* and dye *j*, (*AD*)*_ij_*, gives the following model

$$\begin{array}{l} y_{ijkg}=\mu +A_{i}+D_{j}+{\left(AD\right)}_{ij}+G_{g}\\ +{\left(AG\right)}_{ig}+{\left(VG\right)}_{kg}+{ɛ}_{ijkg}, \end{array}$$

which clarifies that the term represents an effect of the experiment and not an effect of variety.

## 3 Example

In order to study the consequences of Statement 1 and 2, we have reanalyzed the data used in Kerr. Samples of mRNA from human liver tissue and from muscle tissue were compared. The analyzed data contains 1286 clone identifiers representing 1274 genes. The purpose of the experiment was to test whether the clone identifiers (from now on called genes) are differentially expressed between the two types of tissues. The experiment was designed as a latin square (i.e. dye-swap). Model (1) was fitted to the data, and the analysis of variance is given in Table 1, which is an extended version of Table 3 in Kerr. The analysis is performed assuming array effect fixed as done in Kerr. The variety effect and the interaction effect of variety × gene seem to be significant, but due to lack of normality the p-values in the table are only approximate values.

In order to see the difference between the hypotheses in (2) and (3), we carried out the tests in both situations. Due to the deviation from normally distributed errors, we (and Kerr) applied a bootstrap analysis of the residuals (Efron and Tibshirani, 1986), instead of the usual confidence intervals which are based on the assumption of normally distributed errors. We produced a new set of observations by resampling, with replacement, from the original residuals. The new observations were then given by

$$\begin{array}{l} y_{ijkg}^{*}=\hat{\mu }+{\hat{A}}_{i}+{\hat{D}}_{j}+{\hat{V}}_{k}+{\hat{G}}_{g}\\ +{\left(\hat{AG}\right)}_{ig}+{\left(\hat{VG}\right)}_{kg}+{ɛ}_{ijkg}^{*}, \end{array}$$

where the parameters are the estimated parameters from the original fit of the model, and ε*_ijkg_ are drawn independently from $\sqrt{4m/(m-4)}$ ε̂, where *m* is the number of genes and ε̂, are the original residuals. We rescale the original residuals to produce a distribution with the same variance as the true residuals (Kerr). Based on 1000 bootstrap data sets we computed 95% confidence intervals for the differences

$${\left(VG\right)}_{1g}-{\left(VG\right)}_{2g},$$

and for the differences

$$E(\bar{y}.._{1g}-\bar{y}.._{2g})=(V_{1}-V_{2})+({\left(VG\right)}_{1g}-{\left(VG\right)}_{2g}).$$

If the confidence interval does not contain zero, the gene is DE, and either the liver tissue or the muscle tissue have greater expression, depending on the magnitude of the difference. We have not considered the problem of multiple comparisons, and hence the problem of adjusted p-values, neither have Kerr. The results are shown in Table 2. The tests of the two hypotheses conclude differently for 3.1% of the genes. If testing the hypotheses in (2), 17 genes would wrongly (compared to tests of the hypotheses in (3)) be denoted as DE, while 23 genes would wrongly be denoted as EE.

## 4 Conclusion

Methods to detect DE genes have been introduced and applied to microarray data. Among others, Kerr states that “changes in gene expression across experimental samples are captured in the variety × gene interaction terms of the ANOVA model”. If this is correct, the interaction terms can be studied to test the hypothesis of differential expression for individual genes. In this paper we have studied the use of analysis of variance models and hypotheses based on these models, and possible weaknesses of these hypotheses.

We have shown that in situations where there is effect of variety, it is not enough to examine the interaction between variety and gene. This interaction effect will only measure the deviation from the average effect of variety (Black and Doerge, 2002). If the effect of variety increases, more genes become incorrectly denoted as either DE or EE. In situations with no proved significant effect of variety, it is enough to study only the interactions. However, it is not to be recommended, since the test of variety effect may be incorrectly accepted.

## Figures and Tables

**Table 1:** Analysis of variance for the microarray experiment.

Source	DF	SS	MS	F	P
Array	1	92.34	92.34	1433.87	<0.001
Dye	1	0.74	0.74	11.56	0.001
Variety	1	2.97	2.97	46.11	<0.001
Gene	1285	1885.89	1.47	22.79	<0.001
Array × Gene	1285	160.01	0.12	1.93	<0.001
Variety × Gene	1285	1357.28	1.06	16.40	<0.001

Error	1285	82.75	0.0644		

Total	5143	3581.99			

**Table 2:** Number of genes categorized to DE (either Liver < Muscle or Liver > Muscle) or EE by tests of the hypotheses in (2) and (3) respectively.

	(*V*_1_ − *V*_2_) +((*VG*)_1_*_g_* − (*VG*)_2_*_g_*)			
(*VG*)_1_*_g_* − (*VG*)_2g_	Liver < Muscle	Liver = Muscle	Liver > Muscle	
Liver < Muscle	374	0	0	29.1%
Liver = Muscle	23	659	0	53.0%
Liver **>** Muscle	0	17	213	17.9%
	
	30.9%	52.6%	16.5%	1286
